# German National Case Collection for familial pancreatic Cancer (FaPaCa) - acceptance and psychological aspects of a pancreatic cancer screening program

**DOI:** 10.1186/s13053-018-0100-6

**Published:** 2018-11-29

**Authors:** Frederike S. Franke, Elvira Matthäi, Emily P. Slater, Christoph Schicker, Johannes Kruse, Detlef K. Bartsch

**Affiliations:** 1 Department of Visceral-, Thoracic- and Vascular Surgery, National Case Collection for Familial Pancreatic Cancer (FaPaCa), Philipps-University Marburg, Baldingerstrasse, 35043 Marburg, Germany; 2 Department of Psychosomatic Medicine and Psychotherapy, Philipps-University Marburg, Baldingerstrasse, 35043 Marburg, Germany

**Keywords:** Familial pancreatic cancer, Screening program, Acceptance of screening, Psychological aspects of screening

## Abstract

**Background:**

Pancreatic cancer screening is recommended to individuals at risk (IAR) of familial pancreatic cancer (FPC) families, but little is known about the acceptance of such screening programs. Thus, the acceptance and psychological aspects of a controlled FPC screening program was evaluated.

**Methods:**

IAR of FPC families underwent comprehensive counseling by a geneticist and pancreatologist prior to the proposed screening. Participating IAR, IAR who discontinued screening and IAR who never participated in the screening program were invited to complete questionnaires to assess the motivation for participating in surveillance, cancer worries, structural distress and experiences with participation. Questionnaires were completed anonymously to receive most accurate answers.

**Results:**

Of 286 IAR to whom pancreatic ductal adenocarcinoma (PDAC) screening was recommended, 139 (48.6%) IAR regularly participated (group 1), 49 (17.1%) IAR (group 2) discontinued screening after median 1 (1–10) screening visits and 98 (34.2%) IAR (group 3) never underwent screening. The overall response rate of questionnaires was 67% (189/286) with rates of 100% (139 of 139 IAR), 49% (29 of 49 IAR) and 23.4% (23 of 98 IAR) for groups 1, 2 and 3, respectively. At least 93% of IAR felt adequately informed about the screening program after initial counseling. However, only 38.8% received knowledge of or the recommendation for PDAC screening by physicians. The reported cancer-related distress and the fear of investigations were highest in group 1, but acceptably low in all three groups. The main reasons to discontinue or not to participate in screening were the time efforts and travel costs (groups 2 and 3 48,7%).

**Conclusion:**

Less than 50% of IAR regularly participate in a proposed PDAC screening program, although the associated psychological burden is quite low. Physicians should be educated about high risk PDAC groups and screening recommendations. Time and travel efforts must be reduced to encourage more IAR to participate in a recommended screening.

## Introduction

Pancreatic ductal adenocarcinoma (PDAC) is the fourth leading cause of cancer-related deaths in Germany. Despite tremendous efforts made in research, the overall five-year survival of PDAC still remains less than 5%. The annual incidence and mortality of PDAC are almost equal, largely because of late diagnosis and aggressive tumor biology [1, 2]. Early detection at a premalignant stage would offer options for curative therapy. Therefore, screening programs for individuals at high risk (IAR) have been recommended [3].

IAR are defined as individuals with a greater than five-fold risk of developing PDAC. An increased PDAC risk occurs in hereditary syndromes such as Peutz-Jeghers-Syndrome (PJS) and familial atypical multiple mole melanoma (FAMMM), in hereditary pancreatitis and cystic fibrosis and in the setting of the familial pancreatic cancer (FPC) syndrome. FPC accounts for approximately 3–5% of all PDAC cases [4, 5] and 80% of hereditary PDAC cases (11). Families with at least two first-degree relatives (FDR) with PDAC not fulfilling the criteria of another hereditary tumor syndrome are defined as FPC. The life-time risk of developing PDAC in FPC ranges between 10 and 40% depending on the number of affected first-degree relatives [6]. A first-degree relative is defined as a close blood relative that includes the individual’s parents, full siblings, or offspring.

In past years, screening programs for PDAC have been established in the USA and several European countries. These include the North American National Familial Pancreatic Tumor Registry (NFPTR), the German National Case Collection of Familial Pancreatic Cancer (FaPaCa) and the European Registry of Hereditary Pancreatitis and Familial Pancreatic Cancer (EUROPAC) [7–10]. A multidisciplinary approach using endoscopic ultrasonography (EUS) and magnetic resonance imaging (MRI) with magnetic resonance cholangiopancreatography (MRCP) is considered to be the most effective [11]. Biomarkers have been extensively studied, but none is yet validated for clinical use [12]. Screening programs are usually offered to families with 2 or more affected individuals and begin at the age of 40 or 10 years below the youngest age of onset [13–18], even though actual studies have shown the diagnostic yield is significantly higher in IAR older than 50 years of age [19, 20]. It is recommended to perform screening every 12 months [21, 22].

Prospective observational studies have shown that PDAC screening of IAR can detect relevant precursor lesions and early PDACs and thus might be effective [14, 16, 18–20, 23]. However, despite the diagnostic yield, it is also of high relevance to evaluate the acceptance and the psychological aspects of surveillance. The impact of surveillance on psychological functioning has been studied to a large extent in familial colorectal and breast cancer [24–28]. However, little is known about psychological aspects and factors affecting the participation in screening programs for PDAC, since only five studies have so far analyzed the psychological effects of PDAC screening [29–33].

PDAC screening differs greatly from other cancer screening programs such as those for breast cancer or colorectal cancer with respect to the poor prognosis of PDAC, the lack of reliable screening methods and the relatively high morbidity of potentially preventive or curative surgery [34]. Considering these facts it is of utmost importance to evaluate the acceptance and the psychological effects of a standardized PDAC screening program in a large cohort of IAR.

## Patients and methods

### Individuals at risk (IAR)

The German National Case Collection for familial Pancreatic Cancer (FaPaCa) was established in 1999 to prospectively collect FPC families [9, 10]. Screening results of the majority of IAR, in particular MRI, EUS and pathological findings, were recently reported [19, 20]. As previously suggested, the diagnosis of FPC was based on the presence of two or more FDR, with a confirmed diagnosis of PDAC, and without evidence of any other inherited tumor syndrome [35]. FPC families were included based on a three-generation family pedigree and confirmation of all cancer diagnoses in the family by review of medical and pathological records, death certificates, and by revision of the pathology slides whenever available. Members of families fulfilling the criteria of FPC were offered mutational analyses of the *BRCA1/2, PALB2* and *CDKN2a* genes as previously described [20, 36, 37].

All IAR from the above defined FPC families were offered participation in board-approved PDAC screening programs that were conducted exclusively at the University Hospital Marburg between July 2002 and December 2015. The following individuals were classified as IAR and encouraged to participate in PDAC screening:

- members of FPC families who are FDR of affected patients
- mutation carriers of a *BRCA1/2, PALB2* and CDKN2a germline mutation with at least one affected PDAC patient in the family, independent of the degree of relationship

IAR were considered to be at high risk, if they were members of a family with 3 or more affected relatives with PDAC, and at moderate risk, if they were members of a family with 2 affected FDR. During initial comprehensive counseling by a geneticist and a pancreatologist, all IAR were offered PDAC screening with EUS and MRI surveillance as part of a board certified research protocol. In this counseling the yet unproven effectiveness of screening, the possibility of false negative or false positive results, as well as a possible cancer diagnosis or findings of undetermined significance were explained.

IAR were selected for PDAC screening if they provided informed consent to participate in the study. Screening started 10 years before the youngest age of onset in the family or by the age of 40 years, whichever occurred earlier. The registry as well as the prospective screening program were approved by the local Ethics Committees (36/1997; last Amendment Sept. 2010).

### Screening protocol

The surveillance program included annual screening with MRI with MRCP (Magnetic resonance cholangiopancreatography) and EUS (endoscopic ultrasound) between 2002 and 2010. The screening protocol was modified based on our initial analyses that revealed a relatively low diagnostic yield of potentially relevant lesions [10, 38]. Since January 2011 follow-up imaging consisted of annual MRI with MRCP and EUS only every third year or when suspicious alterations were detected by MRI. The screening visit was one day if only MRI was performed and in the case of MRI as well as EUS examinations. Prior to the imaging procedures, all IAR were examined by a physician, who also updated the medical and familial history.

If the diagnostic workup was uneventful at baseline, a follow-up examination was recommended after 12 months. When a pancreatic lesion suspicious of malignancy was identified in any of the imaging modalities, the findings were reviewed by an interdisciplinary board consisting of surgeons, radiologists, gastroenterologists and pathologists to determine further management, either intensified surveillance possibly including FNAC (Fine-needle aspiration cytology) or surgery.

### Questionnaire study

All counseled individuals from the FaPaCa registry were invited to complete a questionnaire between 02/2014 and 02/2015. All regular participants of the PDAC surveillance study (group 1) received a letter of invitation with the questionnaire at the time of screening. IAR who discontinued the screening program (group 2) and IAR who never participated in screening (group 3) received a letter of invitation with the questionnaire between March 2014 and February 2015. IAR were asked to complete questionnaires anonymously to receive most accurate answers.

### Measurements

A systematic literature search was conducted using PubMed to obtain an overview of the current state of research. Medical subject headings were [Pancreatic Cancer] AND [Screening] AND [Distress] AND [psychological impact/burden].

Individual sociodemographic and clinical data, including marital status, offspring, level of education, personal cancer history, family cancer history and surveillance results, could not be analyzed, since the questionnaires were completed anonymously to receive most accurate answers. The only data collected were age, sex and genetic background (moderate and high risk FPC, mutations).

We designed similar questionnaires for groups 1 and 2 to assess the motivation towards screening, screening-related cancer-related worries, specific and general distress, the communication with the physicians and experiences with the interventions EUS and MRI during the screening program. Participants were asked to select from a checklist their motive(s) for undergoing PC surveillance [29]. Attitudes towards and experiences with participation in PC surveillance was assessed by a validated 16-item questionnaire already used by Stigglebout et al. [29] and Harinck et al. [39] which was only modified with regard to the wording of questions. The questionnaire comprised four subscales assessing communication with the physician, reassurance, nervous anticipation, and specific perceived disadvantages. Furthermore, specific questions about experiences with each of the surveillance interventions (EUS and MRI) were asked in adaptation to the study of Harinck et al. [30]. The perceived benefits and barriers to PDAC surveillance were assessed with six questions adapted from previous work [30, 39]. The questionnaire for group 2 contained an additional question to evaluate the reasons for leaving the screening program. Questionnaires were completed by the IAR after having attended at least one examination visit.

The third questionnaire designed for individuals who never participated in the surveillance program (group 3) contained 3 additional questions to explore the reasons for not participating in the recommended screening program.

### Statistics

Descriptive statistics were generated to report on the patients’ characteristics, their experiences with the surveillance interventions, and to document the prevalence of psychological distress. All analyses were conducted using GraphPad Prism version 6.0 for Macintosh, GraphPad Software, San Diego, CA, USA. *p* values < 0.05 were considered to be statistically significant.

## Results

PDAC screening was recommended to 280 IAR during initial comprehensive counseling. Of those 187 (67%) underwent at least one screening visit, 139 (49.6%) IAR regularly participated (group 1), 48 (17.1%) IAR (group 2) discontinued screening and 93 (33.2%) IAR (group 3) never underwent screening. The characteristics of the 3 IAR groups, including gender, age at first counseling and prevalence of FPC or syndromic PDAC were not statistically different. The number of high risk or medium risk IAR was significantly different between groups 1 and 3 (*p* = 0.0031). Concerning the travel distance to the screening visit, we also observed a statistically significant difference between groups 1 or 2 and group 3 (*p* < 0.0001 and *p* = 0.0055), respectively. The number of screening visits was higher in group 1 (see Table 1).

**Table 1:** IAR characteristics (*n* = 286)

	Group 1 (*n* = 139)	Group 2 (*n* = 48)	Group 3 (*n* = 93)	*p*-value
Gender (m/f)	59/80	19/29	40/53	0.9218
Age at 1st counseling, median range	47 years (27–72 yr.)	44 years (27–63 yr.)	49 years (20–73 yr.)	0.1616
Distance to screening visit	Median 190 km	Median 190 km	Median 300 km	1 + 2: 0.3816
< 50 km	6	0	2	1 + 3:< 0.0001
50–100 km	18	4	2	2 + 3: 0.0055
100–200 km	56	21	20	
>200Km	59	23	69	
FPC	127	45	84	0.7882
Syndromic PC				
- p16	0	0	0	
- BRCA1/2	9	1	8	
- PALB2	3	2	1	
- Other	0	0	0	
Number of high risk IAR	74	18	31	1 + 2:0.0671
Number of medium risk IAR	65	30	62	1 + 3:0.0031 2 + 3:0.7096
Underwent surveillance with:			none	
MRI + EUS	139	41		
MRI only	0	7		
EUS only	0	0		
Number of screening visits, median (range)	4 (2–12)	2 (1–8)	none	
Mean value	5.0	2.6		

Overall, 187 of 280 (67%) of IAR completed the questionnaires. Return rates were 100% (139 of 139 IAR), 60% (29 of 48 IAR) and 25% (23 of 93 IAR) for groups 1, 2 and 3, respectively. In group 3 the invitation letter and questionnaire could not be delivered to 22 of 93 (24%) IAR, as these individuals had changed their addresses without informing the registry office. Therefore, the actual response rate in this group was 32% (23 of 71), but still much lower than in groups 1 and 2.

The results of group 1 regarding the recommendation or knowledge of the PDAC screening program are shown in Table 2. More than 50% of IAR were informed about PDAC screening by friends or relatives (*n* = 57, 41%) or through their own research on the internet (*n* = 22, 15.8%), whereas 40.3% were informed by their personal/hospital physicians (*n* = 35, 25.2%) or genetic counselors (*n* = 21, 15.1%).

**Table 2:** Knowledge about the FaPaCa-Screening program (group 1, *n* = 139)

Source	n	%
Recommended by personal physician	14	10%
Recommended by relatives/friends	57	41%
Recommended by hospital physicians	21	15.1%
Recommended by genetic counselor	21	15.1%
Own research, including the Internet	22	15.8%
No comment	4	3%
Total	139	100%

Motivation towards screening, cancer worries and general distress related to PDAC screening are shown in Table 3. From 93 to 96% of IAR considered themselves to be comprehensively informed about the PDAC screening program. Over 90% of group 1 and 2 IARs considered it important to be informed about their health status compared to only 70% of group 3 (*p* < 0.0001). Thoughts of a potential PDAC were experienced as a burden by 37% of group 1 IAR, 10% of group 2 IAR and 30% of group 3 IAR. The confrontation with the disease of relatives during the screening program was considered burdensome by 26, 14 and 9% of IARs of group 1, 2 and 3, respectively. In group 1 58% of IAR were afraid that the examination would show a suspicious result compared to only 10 and 17% of groups 2 and 3, respectively (*p* < 0.0001). It is of note, that group 2 reported a lower frequency of psychological distress than IAR in group 1. Only 7% of group 2 IAR experience psychological stress before a follow-up visit compared to 25% of group 1. However, 89% (124/139) of group 1 IAR reported that the annual screening gives them a feeling of reassurance and 93% (129/139) reported that the perceived advantages outweigh the disadvantages of PDAC screening (data not shown).

**Table 3:** Motivation, cancer worries and general distress of IAR according to questionnaires (excerpt)

Question	Group 1 (*n* = 139) yes/no/no c.	Group 2 (*n* = 29) yes/no/no c.	Group 3 (*n* = 23) yes/no/no c.	*P*-value
Is it important for you to be informed about your health	132 (95%)/7 (5%)/0	27 (93%)/ 0/2 (7%)	16 (70%)/3 (13%)/4 (17%)	1 + 3:< 0.0001 2 + 3:0.0532 1 + 2:0.004
Have you been informed comprehensively about the screening program?	134 (96%)/5 (4%)/0	27 (93%)/2 (7%)/ 0	22 (96%)/1(4%)/0	1 + 3: 1 2 + 3: 1 1 + 2: 0.3472
Is the thought of a possible PDAC a burden?	51 (37%)/80 (58%)/8 (5%)	3 (10%)/24 (83%)/2 (7%)	7 (30%)/16 (70%)/0	1 + 3:0.3642 2 + 3: 0.1018 1 + 2: 0.0213
Is it a burden to you to be confronted with death/disease of your relative(s)?	36 (26%)/ 102 (73%)/ 1 (1%)	4 (14%)/ 24 (83%)/1 (3%)	2 (9%)/ 21 (91%)	1 + 3: 0.1746 2 + 3: 0.5515 1 + 2: 0.1960
Are you afraid that the examination will show a suspicious result?	81 (58%)/51 (37%)/7 (5%)	3 (10%)/0/26 (90%)	4 (17%)/19 (83%)/ 0	< 0.0001
Is the annual screening too frequent?	7 (5%)/132 (95%)/ 0	7 (24%)/21 (73%)/ 1 (3%)	4 (17%)/19 (83%)/0	1 + 3: 0.052 2 + 3: 0.7338 1 + 2: 0.0027
Is the effort (travel costs, time, etc.) too high?	12 (9%)/121 (87%)/6 (4%)	8 (28%)/21 (72%)	11 (48%)/12 (52%)/0	1 + 3:< 0.0001 2 + 3:0.1571 1 + 2:0.0106

no c. – no comment

The annual screening interval was considered to be too frequent by 5% IAR of group 1, 24% IAR of group 2 (*p* = 0.0027) and 17% IAR of group 3 (*p* > 0.05). The screening effort with regard to time and travel cost was considered to be too high by 9% IAR of group 1 compared to 28% IAR of group 2 and 48% IAR of group 3 (*p* = 0.01).

The experience of group 1 IAR with the communication and the course of PDAC screening is shown in Table 4. The communication with the study office and the course of screening was judged to be very good or good by 85 and 86% of IAR, respectively. However, 7.1% (4/56) of group 1 IAR were unsatisfied with the communication. Main criticisms were long waiting times for the closing remarks (15/139, 10.8%) and delays in receiving the medical report (13/139, 9.3%) (data not shown). Only 6.4% (9/139) of group 1 IAR were unsatisfied with the course of screening, mostly due to the delay of MRI and EUS examinations. Only 12% (17/139) of IAR were afraid of the examinations, mostly the EUS and the MRI due to claustrophobia. Of group 1 IAR 35 of 139 (25%) would prefer a special psychological counseling during the screening visit (Table 4). In addition, 33% of IAR would prefer to perform the screening visit in a hospital closer to home (data not shown).

**Table 4:** Experienced communication and PDAC screening course - excerpt of questionnaire responses of group 1 IAR (*n* = 139)

Question	Yes (%)	No (%)	No comment (%)
Communication			
Do the physicians have enough time for you?	108 (78%)	18 (13%)	13 (9%)
Are you able to discuss all things that are of concern to you with your physician?	115 (83%)	11 (8%)	13 (9%)
Were you satisfied with the communication with the study office?		n.a.	
Very good	12 (8.3%/21.4%)		
Good	33 (23.7%/58.9%)		
acceptable	7 (5.0%/12.5%)		
poor	4 (2.9/7.1%)		
no comment	83 (59%)		
Would you prefer special psychological counseling during the screening visit?	35 (25%)	100 (72%)	4 (3%)
Course of screening			
Were you afraid of the examination itself?^a^	17 (12.2%)	114 (82%)	8 (5.7%)
MRI	5 (3.6%)		
EUS	9 (6.4%)		
Blood drawing	3 (2.1%)		
How would you rate the course of screening?		n.a.	
Good	61 (44%/62%)		
acceptable	26 (19%/26%		
unsatisfied	12 (9%/12%)		
no comment	40 (28%)		

*- multiple answers were possible; n.a.- not applicable

The main reasons why group 2 and 3 IAR decided to discontinue or not to participate in the proposed PDAC screening are listed in Table 5. IAR gave time effort and travel expenses (48.7%) as well as the fear of the diagnostic procedures and/or their results (33.3%) as the main reasons not to participate in a regular screening program.

**Table 5:** Main reasons to discontinue or not to participate in PDAC screening – excerpt of questionnaire responses of IAR groups 2 and 3 (18 IAR of group 2 and 21 of group 3 stated their reasons)

Reasons^a^	N (%)
too much effort (time, travel expenses)	19/39 (48.7%)
fear of the diagnostic procedures and/or their results	13/39 (33.3%)
the annual follow-up is too frequent	10/39 (25.6%)
miscellaneous (received a bill (1x), wait time too long (1x), received no medical report (1x), death (2x))	9/39 (23.0%)
to be confronted with potential disease is too burdensome	7/39 (17.9%)
feel too old	6/39 (15.4%)
thought the program was terminated	5/39 (12.8%)
no interest, have no complaints	4/39 (10.2%)

^a^**-** multiple answers were possible

The questionnaire study encouraged 13 of 29 (45%) respondents of group 2 and 8 of 23 (34%) of group 3 to continue with the screening program (data not shown).

## Discussion

A recent consensus conference (Cancer of the Pancreas Screening study [CAPS] summit) stated that IAR for the development of PDAC should be screened by a multidisciplinary approach combining screening and treatment at high-volume centers, preferably within research studies [35]. This precondition was realized in the present study. The CAPS consensus [35], as well as several other studies suggested that PDAC screening of IAR by annual MRI and EUS is effective with regard to the diagnostic yield [10, 14, 17–21] as well as to cost-effectiveness [40]. Despite a cost-effective diagnostic yield, the success of any screening program is strongly related to its acceptance, which depends on the associated psychological aspects. Ultimately, a surveillance program can only be successful, if IAR participate and adhere to the program. Acceptance of PDAC screening and its associated psychological aspects, however, are still an understudied area. A recent study has shown that receptivity towards screening was higher among PDAC family members relative to controls and that receptivity was greater for less invasive methods such as a blood test compared to MRI or EUS [41]. The present study is the first to evaluate the reasons for not participating in a recommended board-approved PDAC screening program besides evaluating the participation rate of comprehensively counseled IAR. The rate of regular annual participation was only 49.6%, which was much lower than the 67% rate reported by a previous North American study [42]. This might be the result of limited knowledge of PDAC screening in our IAR cohort, since a generally low level of knowledge regarding PDAC screening has been reported for IAR of FPC families, despite their desire for this information [43]. This reason, however, seems to be negligible in the present cohort, as 93% of participants who discontinued screening and 96% of non-participants felt comprehensively informed about the screening program. Nevertheless, in the present study 56.8% of IAR were informed about PDAC screening before their first specific counseling in our institution by friends, relatives or their own internet searches compared to 40.2% by personal/hospital physicians or genetic counselors. This is an important fact, since it has been shown that the motivation to undergo a particular screening program or test is strongly related to whether the test is recommended by a physician [43]. Therefore, physicians should be educated about high risk PDAC individuals and recommendations for screening and surveillance of these individuals.

The motivation to undergo PDAC screening is also related to cancer worries, degree of invasiveness, costs and comfort levels [43]. Five previous studies on PDAC screening concluded that participation in PC screening programs does not lead to increased psychological distress, nor to increased cancer worries or general distress [29–31, 33]. It is of note that a recent follow-up PDAC screening study reported a decrease of cancer worries over a course of three years [30]. In another study younger individuals showed a significant decrease in cancer-related intrusive thoughts, cancer-related avoidant thoughts, and cancer worry over time [33]. In our cohort of participants the level of cancer worries appeared also acceptable, since 63% of IAR in group 1, 90% of IAR in group 2 and 70% of IAR in group 3 experienced thoughts of a possible PDAC not as a burden (Table 3). The confrontation with the disease of relatives was seen as a burden to 26%, whereas 58% were afraid of a suspicious examination result. In addition, 25% of participating IAR would prefer an additional psychological counseling during the screening visit. As in previous studies [29–31] the advantages of PDAC surveillance outweighed the disadvantages for the majority of participating IAR (93%) and 89% had a feeling of reassurance by the annual screening.

The present study is the first also to evaluate the groups of participants who discontinued screening and non-participants. It is of note that cancer worries were less frequent in these groups compared to regular participants (see Table 3). This finding might reflect lack of awareness regarding familial risk, a general belief that worry is unproductive, illusion of unique invulnerability [44] or a sense of futility in worrying about this deadly disease in the groups of participants who discontinued screening and non-participants. The last point is especially important for PDAC in contrast to most other gastrointestinal cancers, since there are currently no reliable screening tools to detect PDAC or, even better, its high grade precursor lesions. In addition, no single factor - other than cessation of cigarette smoking – is known that can reduce risk, nor is there any proven chemopreventive strategy.

There might be additional, may be even more important reasons for discontinuing or not participating in PDAC screening, in particular the experience with or fear of the examinations, costs and comfort levels [43]. Fear of the examination was one of the three main reasons in our cohort, since 21% of participants who discontinued screening and 17% of non-participants gave fear of the diagnostic procedures as the reason (data not shown). Thus, it worth thinking about more education and support of potential participants*.* Poor communication with the study office and/or suboptimal course of screening visit was not a significant point, since only 4 IAR (7.7%) gave this reason for not participating. Clearly, travel effort and expenses were the major factors, since 49% of IAR stated these as the main reasons not to participate in screening. This is in concordance with the wishes of 36% of regular participants who would prefer the screening visit in a nearby hospital. Thus, it should be the goal for the future to establish more specialized centers with board-approved PDAC screening programs to encourage more IAR to participate.

The present study has several limitations. First, the response rates among the three groups analyzed differ significantly, which potentially introduced significant bias. Second, individual sociodemographic and clinical variables could not be analyzed, since questionnaires were completed anonymously to receive most accurate answers. Third, IAR with BRCA1/2, PALB2 or CDKN2A mutations were not excluded from the study. These IAR may have greater familiarity with cancer screening tests and their attitudes may differ from other FPC family members in important ways. Fourth, this was a cross-sectional study and over time changes with regard to cancer worries, distress levels and experiences with the screening program could not be assessed. Despite these limitations some strengths are important to note, in particular, its focus on PDAC, which remains the deadliest gastrointestinal cancer. The present study is one of the very few psychosocial studies involving a relatively large sample of IAR from FPC families and, for the first time, a group of participants who discontinued screening as well as one of non-participants that were also evaluated. The importance and timeliness of the study should not be understated, since the incidence of PDAC is continuing to rise and effective screening methods are the focus of ongoing translational research.

## Conclusion

In summary, only about 40% of IAR qualifying for PDAC screening received knowledge or recommendations of PDAC screening from their physicians and only 50% of counseled IAR regularly participate in PDAC screening. Cancer worries and psychological stress associated with PDAC screening appear acceptable. Time effort and travel expenses, however, were stated by IAR to be the main reasons to discontinue screening or not to participate at all. Physicians should be educated about high risk PDAC groups and screening recommendations. Time and travel efforts should be reduced to encourage more IAR to participate in a recommended PDAC screening.

## Abbreviations

CAPS: Cancer of the Pancreas Screening study summit
EUROPAC: European Registry of Hereditary Pancreatitis and Familial Pancreatic Cancer
EUS: Endoscopic ultrasound
FAMMM: Familial Atypical Multiple Mole Melanoma Syndrome
FAP: Familial adenomatous polyposis
FaPaCa: German National Case Collection of familial pancreatic cancer
FDR: first-degree relative
FNAC: Fine-needle aspiration cytology
FPC: Familial pancreatic cancer
HBOC: Hereditary breast–ovarian cancer syndromes
HNPCC: Hereditary nonpolyposis colorectal cancer or Lynch syndrome
IAR: Individuals at risk
MRCP: Magnetic resonance cholangiopancreatography
MRI: Magnetic resonance imaging
NFPTR: The National Familial Pancreas Tumor Registry
PDAC: Pancreatic ductal adenocarcinoma
PJS: Peutz-Jeghers Syndrome
